# Toward efficient and high-fidelity metagenomic data from sub-nanogram DNA: evaluation of library preparation and decontamination methods

**DOI:** 10.1186/s12915-022-01418-9

**Published:** 2022-10-08

**Authors:** Chun Wang, Li Zhang, Xuan Jiang, Wentai Ma, Hui Geng, Xue Wang, Mingkun Li

**Affiliations:** 1 Beijing Institute of Genomics, Chinese Academy of Sciences, and China National Center for Bioinformation, Beijing, 100101 China; 2 University of Chinese Academy of Sciences, Beijing, 100049 China; 3 Department of Geriatrics, Peking University First Hospital, Beijing, 100034 China; 4 Beijing Center for Disease Prevention and Control, Beijing, 100013 China; 5 Center for Excellence in Animal Evolution and Genetics, Chinese Academy of Sciences, Kunming, 650223 China

**Keywords:** Metagenomics, Efficiency and Fidelity, Low microbial biomass, Sub-nanogram DNA, Library preparation, *In silico* decontamination

## Abstract

**Background:**

Shotgun metagenomic sequencing has greatly expanded the understanding of microbial communities in various biological niches. However, it is still challenging to efficiently convert sub-nanogram DNA to high-quality metagenomic libraries and obtain high-fidelity data, hindering the exploration of niches with low microbial biomass.

**Results:**

To cope with this challenge comprehensively, we evaluated the performance of various library preparation methods on 0.5 pg–5 ng synthetic microbial community DNA, characterized contaminants, and further applied different in silico decontamination methods. First, we discovered that whole genome amplification prior to library construction led to worse outcomes than preparing libraries directly. Among different non-WGA-based library preparation methods, we found the endonuclease-based method being generally good for different amounts of template and the tagmentation-based method showing specific advantages with 0.5 pg template, based on evaluation metrics including fidelity, proportion of designated reads, and reproducibility. The load of contaminating DNA introduced by library preparation varied from 0.01 to 15.59 pg for different kits and accounted for 0.05 to 45.97% of total reads. A considerable fraction of the contaminating reads were mapped to human commensal and pathogenic microbes, thus potentially leading to erroneous conclusions in human microbiome studies. Furthermore, the best performing in silico decontamination method in our evaluation, Decontam-either, was capable of recovering the real microbial community from libraries where contaminants accounted for less than 10% of total reads, but not from libraries with heavy and highly varied contaminants.

**Conclusions:**

This study demonstrates that high-quality metagenomic data can be obtained from samples with sub-nanogram microbial DNA by combining appropriate library preparation and in silico decontamination methods and provides a general reference for method selection for samples with varying microbial biomass.

**Supplementary Information:**

The online version contains supplementary material available at 10.1186/s12915-022-01418-9.

## Background

High-throughput sequencing has revolutionized the microbiome research, especially that metagenomics reveals microbial information with higher sensitivity than ever before. Microbial communities have been studied in a variety of human body and environment niches, including those with low microbial biomass, such as skin swabs, cerebro-spinal fluid, placenta, Antarctic fields, deep-sea vents, the atmosphere, and fossils. Genomic DNA extracts of those samples can be down to the picogram level. The 16S rDNA amplicon sequencing is widely used, which overcomes the problem of insufficient input material, but leaves functional potential and taxonomic information at the species and strain levels unexplored. Therefore, shotgun metagenomics profiling of microbial composition and functional information is in urgent need for ecosystems with low microbial biomass.

Two strategies can be applied when using sub-nanogram DNA in shotgun metagenomics. The first is to increase the DNA amount before conventional library construction using various amplification methods [1–4], among which whole genome amplification (WGA, i.e., multiple displacement amplification) is the most widely used. WGA has been proposed to cause microbial community distortion [5–7]. However, previous comparisons were based on nanogram quantities of DNA, and it is unclear whether metagenomics with a lower amount of template can benefit more from this technique. The second is to construct a library directly from low-input DNA. Commercial kits could be classified into three different categories based on the DNA fragmentation method, mechanical fragmentation (sonication or nebulization), endonuclease digestion, and Tn5 transposase tagmentation. Of note, metagenomic library construction with as little as 100 fg of DNA using the tagmentation technique has been reported [8]. Although some of the above methods have been examined for metagenomics using sub-nanogram DNA [3, 9], the methods and evaluation metrics utilized in every single study were limited, thus a more comprehensive evaluation with updated techniques (kits) is still warranted.

Besides the technical issue with the library construction, the contaminating microbial DNA from reagents and the environment is another challenge when dealing with low-biomass samples [10–14]. The contaminating reads could outnumber the endogenous microbial reads, leading to a distorted microbial community and false-positive findings from metagenomic studies [15, 16]. There are a few widely used in silico methods to identify contaminating taxa, including (a) methods based on the relative abundance, i.e., filtering taxa below an ad hoc relative abundance threshold or taxa having comparable abundances in true samples and negative controls; (b) the Decontam method, i.e., filtering taxa having frequencies that negatively correlate with input DNA quantity and/or taxa having a higher prevalence in negative controls than in true samples [17]; and (c) the SourceTracker method, which applies a Bayesian approach to estimate the proportion of sequences originated from defined contaminant sources [18]. Some of the methods have been evaluated using 16S rDNA amplicon sequencing data generated from a dilution series of a mock microbial community, revealing that none of the methods was able to completely remove the contaminants [19]. However, the performance of these decontamination methods has not been systematically compared using shotgun metagenomic data, and it is unknown whether it differs among library preparation methods with varied loads and patterns of contaminants.

In this study, we used a well-designed artificial DNA material as a template, which represents a wide range of microbes with broad coverage of GC contents and concentration gradients and has no homology with any known sequences [20]. We aimed to evaluate the performance of different sequencing library preparation and decontamination methods and to provide a reference for method selection toward obtaining efficient and high-fidelity metagenomic data from samples with low microbial biomass.

## Results

### Overview of experiment design

**Fig. 1:**
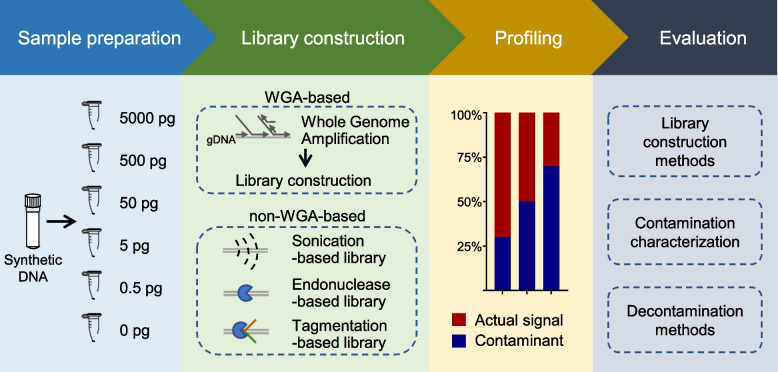
Overview of the experiment design. WGA, whole-genome amplification; gDNA, genomic DNA; Son_N, Nugen Ovation Ultralow System V2 Kit; Son_Q, QIAGEN QIAseq Ultralow Input Library Kit; End_N, NEBNext Ultra II FS DNA Library Prep Kit; End_Q, QIAGEN QIAseq FX DNA Library Kit; Tn5_V, Vazyme TruePrep DNA Library Prep Kit

The synthetic microbial community DNA (sequins), which consists of 83 artificial sequences with the proportion of mass varying from 0.01 to 6.9% (Additional file 1: Table S1), was serially diluted to generate positive samples with 5000 pg, 500 pg, 50 pg, 5 pg, and 0.5 pg DNA as well as negative controls. The samples were then subjected to WGA-based and non-WGA-based library constructions. For the latter, libraries were prepared using five commercial kits capable of handling low-biomass samples, including two kits applying sonication-based fragmentation, i.e., Nugen Ovation Ultralow System V2 Kit (Son_N) and QIAGEN QIAseq Ultralow Input Library Kit (Son_Q); two kits applying endonuclease digestion, i.e., NEBNext Ultra II FS DNA Library Prep Kit (End_N) and QIAGEN QIAseq FX DNA Library Kit (End_Q); and one kit applying Tn5 tagmentation, i.e., Vazyme TruePrep DNA Library Prep Kit (Tn5_V) (Additional file 1: Table S2). Two experimental replicates were performed by different operators to evaluate the reproducibility of the methods. Shotgun sequencing was performed on all libraries, obtaining a median of 16.6 million reads per library (range 7.3–23.2 million, Additional file 2: Fig. S1a). The data from each library was separated into two parts, the synthetic sequins and the contaminants, which were used to evaluate library construction methods and in silico decontamination methods (Fig. 1, see rarefaction curves in Additional file 2: Fig. S1b and c).

### WGA substantially changed the microbial composition with sub-nanogram templates

**Fig. 2:**
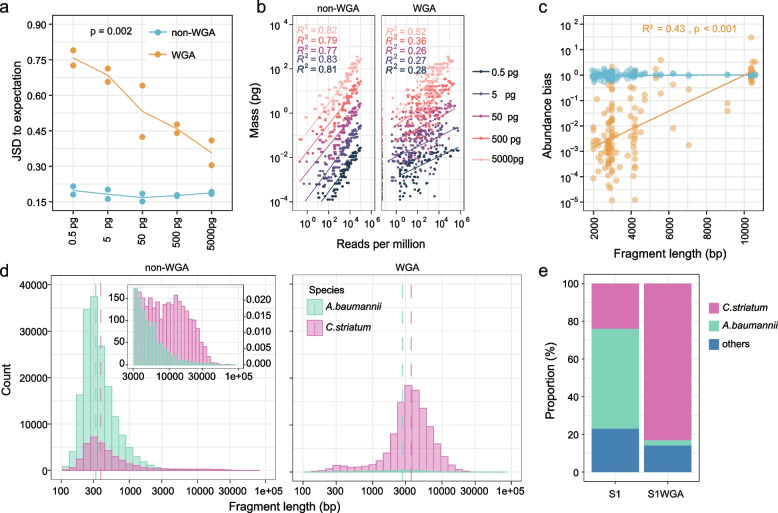
The impact of WGA on metagenomic profiling with sub-nanogram DNA. **a** JSD between the expected and measured composition of sequins. The *p* value from the Wilcoxon test is shown. **b** Correlations between the input mass of sequins components and corresponding numbers of reads per million. Different levels of input amount are indicated by colors; *R*^2^ values from linear regression models are shown, *p* values < 0.001. **c** Correlations between the abundance bias of sequins components and corresponding fragment lengths for libraries with 0.5 pg template. *R*^2^ and *p* values from linear regression models are shown. **d** The length distribution of Nanopore sequencing reads aligned to *A. baumannii* and *C. striatum* in a sputum sample measured with WGA-based (right) and non-WGA-based (left) libraries. Dotted lines indicate the median lengths. **e** Relative abundances of dominant genera in the sputum sample

As expected, WGA elevated the DNA amount from 0.5–5000 pg to 18–34 μg. However, the proportion of reads originating from sequins was less in the WGA-based libraries than that in the non-WGA-based libraries when the template DNA was 50 pg or lower (median 86.0% vs. 99.2%, *p* < 0.01, Wilcoxon test). Meanwhile, the non-designated reads, including microbial and non-microbial contaminating reads and unclassified reads, increased markedly with the reduced amount of template DNA for WGA (Additional file 2: Fig. S2a). Besides, compared to the non-WGA-based libraries, the composition of sequins estimated from the WGA-based libraries was more distorted, especially with low template input, reflected by larger Jensen-Shannon distance (JSD) to the theoretical composition of sequins (*p* < 0.01, Wilcoxon test, Fig. 2a), poorer correlation between the input mass of each sequins component and corresponding read numbers (Fig. 2b), as well as forming a different cluster from theoretical sequins and non-WGA libraries on the principal coordinates analysis (PCoA) plot and heatmap (Additional file 2: Fig. S2b and c). In addition, the JSD between the sequins composition of experimental replicates of WGA-based libraries was greater than that of non-WGA-based libraries, indicating poor reproducibility of WGA in terms of component enrichment (Additional file 2: Fig. S2d).

Interestingly, we found that the fragment length was positively correlated with the magnitude of WGA enrichment bias (*p* < 0.001, linear regression, Fig. 2c), which was represented by the ratio between the abundance of each sequins component in WGA-based libraries and its mean abundance in non-WGA-based libraries. It suggested that the efficiency of WGA might be higher for longer template DNA. This is further supported by Nanopore sequencing data from a sputum sample, where the read lengths of the two dominant taxa rose from 323 to 2703 bp and from 381 to 3606 bp following WGA (Fig. 2d) respectively for *Acinetobacter baumannii* and *Corynebacterium striatum*. Meanwhile, the relative abundance of *C. striatum*, whose DNA template was longer, increased from 15.2 to 86.5% when WGA was implemented (Fig. 2e). Furthermore, we found that WGA treatment led to uneven genome coverage, particularly for 5 pg and 0.5 pg templates (Additional file 2: Fig. S2e and f) and that WGA biased toward DNA fragments with lower GC content (Additional file 2: Fig. S2g and h), which was in line with previous reports [5–7].

Collectively, WGA introduced a substantial amount of contaminants and showed poor fidelity for sub-nanogram templates, making it unsuitable for metagenomic profiling of low-biomass samples.

### Evaluation of non-WGA-based DNA library preparation methods

**Fig. 3:**
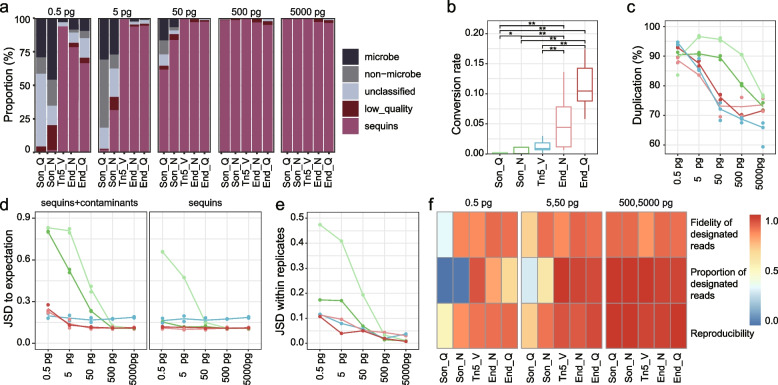
Performance of non-WGA-based DNA library preparation methods. **a** Read composition of raw data. **b** Library conversion rate. *p < 0.05, **p < 0.01, Wilcoxon tests. **c** Duplication rate of sequins. **d** JSD between the expected compositions of sequins and measured profiles including sequins and microbial contaminants (left) or measured sequins profiles (right). **e** JSD between the measured profiles of experimental replicates. **f** Evaluation of methods based on three crucial parameters. Fidelity of designated reads was defined as 1 subtracted by JSD between the expected composition of sequins and measured sequins profiles, and reproducibility was defined as 1 subtracted by JSD between the measured profiles of experimental replicates

We next evaluated the quality of libraries prepared using sub-nanogram templates without the implementation of WGA. First, the proportion of reads originating from sequins were higher than 90% for all methods using 500 pg and 5000 pg templates (94.81–99.77% with median 98.68%), but decreased dramatically when the template load became lower except for Tn5_V, with 93.7%, 78.21%, 66.29%, 1.64%, and 0.06% of total reads assigned to sequins for Tn5_V, End_N, End_Q, Son_N, and Son_Q, respectively, when the template load was 0.5 pg (Fig. 3a). Second, the library conversion rate (the rate between actual library quantity and theoretical library quantity) was the highest using End_N and End_Q, followed by Tn5_V, Son_N, and Son_Q (Fig. 3b). Third, the sonication-based libraries had lower complexity (i.e., higher duplication rate, the ratio of the number of duplicates over the number of total mapped reads) than the other methods (Fig. 3c). Moreover, the insertion size of library constructed with Tn5_V showed the most optimal distribution with a peak range of 292–371 bp, which was probably attributed to the stricter size selection applied in the protocol. In contrast, End_Q, End_N, and Son_Q showed smaller insertion sizes (peak 193–244 bp, 143–210 bp, 139–349 bp, respectively), while Son_N showed a larger insertion size with higher variance (range 181–634 bp, Additional file 2: Fig. S3a).

To test the fidelity of various library preparation methods, we calculated the JSD between the expected sequins composition and the measured profiles, which included both sequins and contaminating microbes. The endonuclease-based data showed the least deviation from the expected composition, followed by the tagmentation-based data, whereas the sonication-based data showed significantly higher deviation for samples with 50 pg or less input. After removing contaminants and only comparing the composition of sequins, we found similar results, with the exception that the performance of Son_N significantly improved (Fig. 3d). The fidelity might be associated with GC content bias, as there was a considerable shift of GC distribution of the reads toward lower GC values compared to the theoretical distribution, especially for the tagmentation-based method (Additional file 2: Fig. S3b). Besides, there was a weak positive correlation between the abundance bias (ratio between measured abundance and the theoretical value) and fragment length for all methods (*p* values < 0.001, linear regression), and the tagmentation-based method showed the highest coefficient of determination (*R*^2^ = 0.14, Additional file 2: Fig. S3c). Meanwhile, no correlation was observed between the length and GC content of sequins components (*p* = 0.76), suggesting that the fragment length may independently influence the fidelity. In addition, the reproducibility of all methods decreased gradually with reduced input, and endonuclease- and tagmentation-based methods performed better than sonication-based methods (Fig. 3e).

Summarizing three crucial metrics, i.e., fidelity of designated reads, the proportion of designated reads, and reproducibility, when there was 5000 or 500 pg input material, the performance of all methods was equally well except that the tagmentation-based method had lower fidelity; when there was 50 or 5 pg input, endonuclease-based methods showed better and more balanced performance on all three metrics than other methods; when there was 0.5 pg input, the tagmentation-based method and one endonuclease-based method (Enz_N) performed better than the other endonuclease-based method (Enz_Q), followed by sonication-based methods (Fig. 3f). Of note, the tagmentation-based method had a significantly higher proportion of designated reads than other methods with 0.5 pg input, suggesting its attractive advantage on samples with very low biomass.

### Characteristics of contaminants introduced by library preparation

Contaminating reads originated from background DNA increased as the input load decreased, and the microbial reads accounted for 3.06–45.97% of total reads when the input load was 0.5 pg (Fig. 3a). The PERMANOVA analysis revealed that library preparation kits explained the largest variance in contaminating microbial composition (*R*^2^ = 36.4%, *p* < 0.001, Additional file 2: Fig. S4a), suggesting that a large fraction of the contaminants was kit specific. Surprisingly, input amount appeared as the second significant explaining factor in the PERMANOVA analysis (*R*^2^ = 15.6%, *p* < 0.001), suggesting that there were some endogenous contaminants in the sequins. We found that the abundance (normalized to total contaminating reads) of five genera, i.e., *Escherichia*, *Gammaretrovirus*, *Citrobacter*, *Mastadenovirus*, and *Shigella* was positively correlated with the input amount (p.adj < 0.05 and *R*^2^ > 0.55 for at least four kits, linear regression, Additional file 2: Fig. S4b), suggesting that they mainly originated from the sequins material and were thus filtered out in the following analyses. The variance explained by the input amount in the PERMANOVA analysis after the filtration reduced to 8.7% (*p* < 0.001).

**Fig. 4:**
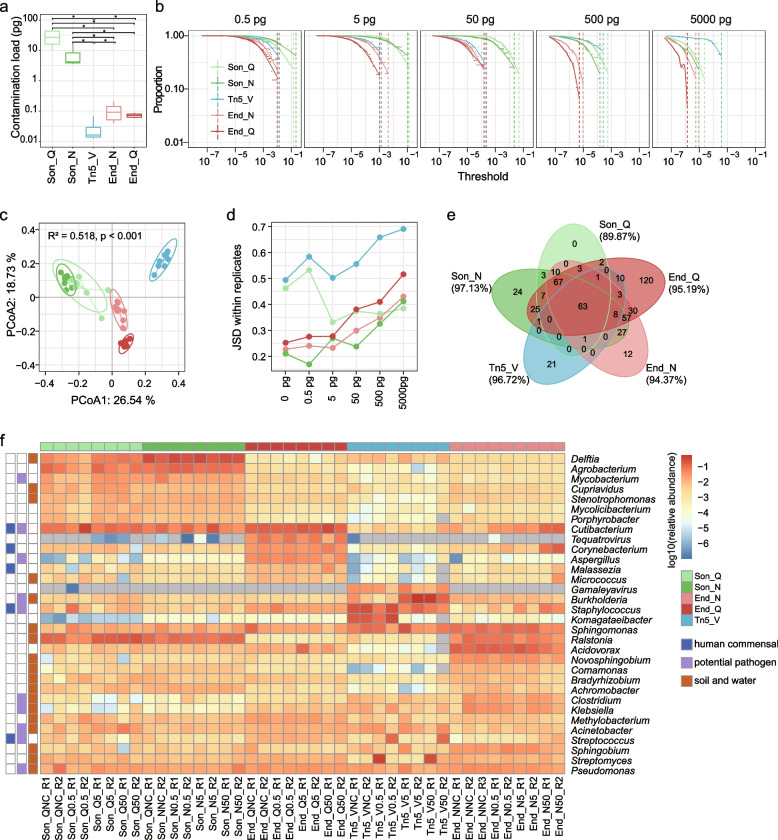
DNA contaminants introduced by library preparation. **a** Quantity of contaminating DNA in each kit. *p < 0.05, Wilcoxon tests. **b** The proportion of remaining contaminants (out of all contaminating reads), after filtering components below certain relative abundances. Dotted lines indicate relative abundances of the most abundant contaminating genera in libraries with each input amount, where the proportion reaches 0. **c** PCoA plot based on JSD of contaminating compositions. *R*^2^ and *p* values from PERMANOVA are shown. Circles indicate 95% confidence. **d** JSD between contaminating compositions of experimental replicates. **e** Venn diagram of core contaminating genera, which had relative abundances higher than 0.1% in at least half of the libraries for each kit. The proportion of core contaminating genera in all microbial contaminants for each kit is shown in brackets. **f** Heatmap showing 32 dominant contaminants, which had relative abundances higher than 1% in at least half of the libraries for each kit. Common niches of the microbes are indicated on the left. In **a**, **c**, and **f**, libraries with 0.5–50 pg input are shown whereas all libraries are used in the rest panels

We quantified the absolute amount of contaminating DNA using sequins as a spike-in control. Sonication-based methods had the highest amount of contaminants, with 15.59 pg for Son_Q (median) and 2.32 pg for Son_N, whereas other methods had fewer contaminants (0.05 pg, 0.04 pg, and 0.01 pg for End_N, End_Q, and Tn5_V, respectively, Fig. 4a). The most abundant contaminating genus of each kit had a relative abundance lower than 0.06% in libraries with 500 pg and 5000 pg input (Fig. 4b, Additional file 2: Fig. S4c), indicating that contamination was not a major concern when the input load was high. As the input decreased to 0.5 pg, the relative abundance of the highest contaminating genus increased to 21.4%, 14.2%, 1.3%, 1.4%, and 1.0% for Son_N, Son_Q, Tn5_V, End_N, and End_Q, respectively, and the proportion of remaining contaminants (out of all contaminating reads) was 91.1%, 87.1%, 76.0%, 65.9%, and 51.5% when setting a relative abundance threshold of 0.1% (Fig. 4b, Additional file 2: Fig. S4c).

Concerning the contaminating microbial composition for each kit, two sonication-based kits (Son_N and Son_Q), End_Q, End_N, and Tn5_V formed four distinct clusters on the PCoA plot (PERMANOVA *R*^2^ = 0.52, *p* < 0.001, Fig. 4c). The JSD between contaminating compositions of experimental replicates generally increased when the input amount was higher, suggesting greater randomness with a reduced contaminating fraction (Fig. 4d). We detected 494 core contaminating genera (with relative abundance higher than 0.1% in at least half of the libraries for each kit) that accounted for more than 89% of contaminating microbial reads in each kit (Fig. 4e, Additional file 1: Table S3). Of these genera, 110 have been found to be contaminants in previous studies [10–14, 21, 22]. Sixty-three core genera were shared by all five kits and accounted for more than 55% of contaminating reads in each kit. End_Q, Son_N, Tn5_V, and End_N had 120, 24, 21, and 12 unique contaminating genera respectively, while Son_Q had no unique ones (Fig. 4e). There were 32 high-abundance contaminants (greater than 1% in at least half of the libraries in each kit), which varied depending on the kit used (Fig. 4f). Among them, many genera are frequently identified in soil and water, e.g., *Acinetobacter*, *Bradyrhizobium*, and *Methylobacterium*. Of note, some genera are commensal microorganisms or potential pathogens that reside in human skin and mucosa, e.g., *Cutibacterium*, *Staphylococcus*, *Corynebacterium*, *Acinetobacter*, *Streptococcus*, and *Klebsiella*, with relative abundances up to 20.4%, 9.4%, 4.1%, 3.2%, 1.8%, and 0.3%, respectively, which could result in erroneous conclusions in human microbiome studies and pathogen detection. Finally, we found that the genome coverage of contaminating microbes was stochastic (Additional file 2: Fig. S4d), making it difficult to distinguish between contaminants and actual signals.

### In silico decontamination methods recovered the real microbial community from high-quality libraries

Three types of most widely used decontamination methods, including filtering taxa with relative abundances less than fivefold or tenfold of that in negative controls (referred to as fivefold-NC and tenfold-NC), the Decontam method [17] (frequency mode, prevalence mode, and *either* mode), and the SourceTracker method [18], were employed to eliminate the impact of contamination. An optimum threshold for the Decontam method was determined as the one that resulted in the best recall with the premise of 100% precision (Additional file 2: Fig. S5a-e). True positives, false negatives, true negatives, and false positives were defined as contaminants accurately detected, contaminants missed, sequins detected as actual signals, and sequins misclassified as contaminants, respectively.

**Fig. 5:**
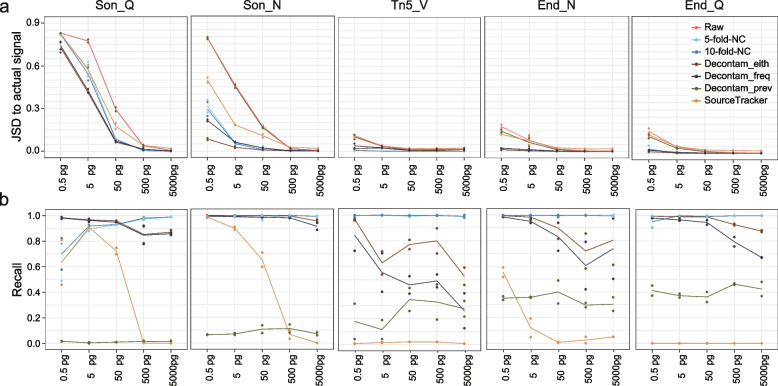
Performance of in silico decontamination methods. **a** JSD between actual signals (i.e., measured sequins profiles) and complete profiles (including sequins and microbial contaminants) with or without applying decontamination methods. **b** The recall of different methods in identifying contaminants

For Tn5_V, Enz_N, and Enz_Q libraries, which had a relatively low level of microbial contaminants (maximum < 10% of total reads, Fig. 3a), the JSDs between the decontaminated profile and the actual signal for Decontam-either, Decontam-frequency, and 5/tenfold-NC were small (< 0.05, Fig. 5a), with the most abundant contaminating genera having a relative abundance of 0.32% in the decontaminated profile, indicating that the above methods were able to recover the real microbial community from contaminated data. The recall for 5/tenfold-NC was close to 1, whereas the recall for Decontam-either and Decontam-frequency declined dramatically with the increased input load (Fig. 5b); however, there was little fidelity loss as the false-negative contaminants had low abundances.

For Son_Q and Son_N libraries that suffered heavy microbial contaminants (0.06–30.89% with a median of 16.69% and 0.06–45.97% with a median of 6.86%, respectively, Fig. 3a), Decontam-either, Decontam-frequency, and 5/tenfold-NC performed equally well when the input loads were 500 pg and 5000 pg, with the JSD between the decontaminated profile and the actual signal close to zero (Fig. 5a). However, the decontamination efficacy of the three methods decreased as the input load became lower despite of high recalls. Among them, Decontam-either was the most effective, followed by Decontam-frequency and 5/tenfold-NC (Fig. 5a, b). Interestingly, although Son_N and Son_Q libraries had similar levels of microbial contaminants when the input load was 0.5 pg, the decontamination methods performed much better on the former data (e.g., JSD for Decontam-either, 0.08 vs. 0.73, Fig. 5a), which could be due to significantly higher consistency of contaminants in Son_N libraries (Fig. 4d, e). Of note, although the best performing method Decontam-either removed at least 95.8% of the contaminating reads for Son_Q libraries with 0.5 pg and 5 pg inputs, there was still a large difference between the decontaminated profile and the actual signal (JSD > 0.5), highlighting the limitation of the current decontamination algorithms.

In addition, Decontam-prevalence and SourceTracker showed poor efficacy of decontamination for most libraries, owing to their low recalls and in part the low precisions of SourceTracker in some libraries (Fig. 5b, Additional file 2: Fig. S5f). Rarefying data to the same sequencing depth (7.3 million reads) did not change the above results.

## Discussion

Metagenomic data with high efficiency, high fidelity, and high reproducibility is essential for the understanding of microbial communities in biological niches with low microbial biomass. We found that constructing libraries directly from sub-nanogram templates was superior to conducting WGA before library preparation. Among the non-WGA-based library preparation methods, we recommend using an endonuclease-based method for a broad range of input loads. If the majority of the samples in a study have microbial biomass as low as 0.5 pg, a tagmentation-based method is recommended due to its high efficiency in generating sequences from designated components, which is probably attributed to its procedure of combining fragmentation and adaptor ligation into one step that resulting in high DNA recovery. Moreover, although current commercial kits designed to deal with ultralow inputs are mostly based on sonication, their performance with less than 500 pg input was quite poor in our evaluation. We suspect that both the extra processing steps required by sonication and the mechanical fragmentation itself induce substantial DNA damage and loss.

Even though suitable library preparation methods can yield a high proportion of designated reads, microbial contaminating reads are unavoidable, thus in silico decontamination is necessary especially for low-biomass samples. We propose using the Decontam method with *either* mode, which filters taxa that show negative correlations with the input load, as well as taxa that are more prevalent in negative controls than in positive samples. The threshold should be chosen with caution to avoid deleting actual signals especially for the latter strategy. After decontamination, high-quality data resembling the real microbial community could be obtained from libraries with less than 10% of total reads attributed to contaminants, but not libraries with heavy and highly varied contaminants. This underscores the need to integrate appropriate experimental and bioinformatics approaches, while also emphasizing the need for improved decontamination algorithms.

We created a standardized dataset with clear labels separating actual signals and contaminants, as well as diversified levels and patterns of contaminants, which will be useful in the development of new decontamination algorithms. We identified about 500 contaminating genera that were mainly introduced by different library preparation kits, and more than half of them had never been reported before, adding new microbes to the reference list of common contaminants. It also uncovered the differences between contaminants using different protocols and laboratories, which has been demonstrated previously by Salter et al. [10]. Therefore, each laboratory should establish its own contaminant databases by constructing libraries using serial dilutions of a mock microbial community or its DNA, and repeat this process on a regular basis, which is crucial for monitoring contaminant levels and profiles, as well as setting a proper threshold for decontamination pipelines.

There are limitations to this study. For the evaluation of library construction methods, we constructed libraries following the instruction of each kit’s manufacturer, which may alter the comparability of methods. For example, double size selection using purification beads was only included in the protocol of Tn5_V, which resulted in an optimal insertion size distribution but an underrated library conversion rate for this kit. Additionally, the evaluation could be improved by including more library preparation methods/kits, comparing different batches of each kit, and determining interlaboratory reproducibility. For the evaluation of in silico decontamination methods, the synthetic DNA material is not able to represent some real-world scenarios, such as the varied complexity of different microbial communities, and the presence of microbial taxa in both positive samples and environmental contaminants. For example, the 5/tenfold-NC method is expected to have reduced precision in real-world studies than evaluated in this study. Besides, well-to-well cross-contamination caused by splashing [23] or bioaerosol is common and can hardly be detected by in silico decontamination methods, and this issue was not considered in this study. Finally, our study did not consider all steps of the metagenomic workflow. The impact of contamination from DNA extraction kits and different quantities of host DNA has yet to be studied.

## Conclusions

Altogether, our study provides a general reference for method selection in metagenomic studies with sub-nanogram microbial DNA. For library preparation, we recommend the non-WGA-based methods, including the endonuclease-based method for a broad range of input loads and the tagmentation-based method for ultra-low input loads around 0.5 pg. For in silico decontamination, we recommend the Decontam method with *either* mode, for which a proper threshold setting is important. By combining appropriate library preparation and in silico decontamination methods, high-quality metagenomic data can be obtained from samples with low microbial biomass.

## Methods

### The mock DNA

Sequins for metagenomics [20] mix version C was kindly provided by Garvan Institute of Medical Research, Australia. It consists of a pool of 83 artificial DNA sequences, including 70 sequences that were selected and inverted from the genomes of 62 species (41 Gram-negative Bacteria, 19 Gram-positive Bacteria, one Archaea, and one parasitic protozoan). The sequences are combined at twofold serial dilutions to encompass a 128-fold molar concentration range. The mixture has eight staggered concentration points, with at least 5 different sequences per point to represent a wide range of GC contents (29.4–71.06%) and lengths (1929–9120 bp). Besides, it contains a trace but unknown amount of 92 byproduct sequences (Additional file 1: Table S1). The concentration of sequins was measured using Qubit 4.0 fluorometer (Life Technologies, Singapore).

### Library construction and shotgun sequencing

For the WGA-based strategy, DNA was amplified using Qiagen REPLI-g WGA kit (Hilden, Germany), purified using 1.8 × Beckman Ampure XP beads (Beckman Coulter, CA, USA), and used for library construction with Vazyme TruePrep DNA Library Prep Kit TD501 (Vazyme, Nanjing, China). For the non-WGA-based strategy, Nugen Ovation Ultralow System V2 (NuGEN Technologies, SC, USA), QIAGEN QIAseq Ultralow Input Library Kit (Hilden, Germany), NEBNext Ultra II FS DNA Library Prep Kit (New England Biolabs, Hertfordshire, UK), QIAGEN QIAseq FX DNA Library Kit (Hilden, Germany), and Vazyme TruePrep DNA Library Prep Kit TD502/TD503 were used following the manufacturer’s instructions. The number of PCR cycles was listed in Additional file 1: Table S2. Sonication of DNA to 350 bp was done using Covaris S2 (Woburn, MA, USA). The library concentration was measured using real-time PCR with primers targeting adaptors, and the library length was measured using Labchip GX Touch Nucleic Acid Analyzer (PerkinElmer, Hopkinton, MA). Shotgun sequencing was performed on Illumina Novaseq 6000 PE150 platform.

High-standard practice was followed to minimize laboratory contamination during library preparation, including performing all experiments in a class 100,000 cleanroom, wearing surgical caps and masks, as well as the application of sterile equipment and pipette tips with filters and ultra-low retention. Molecular biology-grade nuclease-free water (Invitrogen) was used in all processes.

### Library construction and Nanopore sequencing

Genomic DNA was extracted from the sputum sample using the QIAGEN DNeasy PowerSoil Pro Kit (Hilden, Germany). An aliquot of 6 ng DNA was amplified using Qiagen REPLI-g WGA kit and then debranched using T7 endonuclease I (New England Biolabs), while another aliquot of 1.7 ug DNA was directly used for PromethION library preparation. PCR-free libraries were constructed with Native Barcoding Kit EXP-NBD104 (Oxford Nanopore Technologies, Oxford, UK) according to the manufacturer’s instructions. The MinKNOW software v19.10.1 was used to collect raw sequencing data, and Guppy v3.2.4 was used for local base-calling.

### Sequencing data analysis

Illumina raw reads were processed by filtering poly-G sequences and low-quality sequences with fastp v0.20.0 [24] and BBDuk v39.92 (sourceforge.net/projects/bbmap/) and trimming adapters with Trimmomatic v0.36 [25]. Paired-end reads were merged using Flash v1.2.11 [26]. Processed reads were mapped to the sequins reference using BWA v0.7.12-r1039 [27] and summarized using samtools v1.8 [28] to obtain the sequins composition and sequencing coverage. The duplication rate was determined by samtools v1.8, which treats reads with the same 5 prime positions of both reads and read-pairs as duplicate reads. Kraken2 v2.1.2 [29] was used for the taxonomic classification of non-sequins reads at the genus level. SeqKit v0.10.2 [30] was used to measure the GC content and length of reads. ART v2.5.8 [31] was used to generate simulated PE150 sequencing data with at least 40 × coverage for each sequins component.

The library conversion rate was estimated as follows:

$$\mathrm{Conversion}\;\mathrm{rate}\;=\frac{Q_{\mathrm{library}\;}\ast\;R_{\mathrm{designated}}}{Q_{\mathrm{input}\;}\ast\;E_{\mathrm{primer}}^{N_{pcr}}}\\$$

where *Q*_library_ is the library quantity measured by Qubit, *Q*_input_ is the input DNA quantity, *E*_primer_ is a constant 2 representing a 100% primer efficiency, *N*_pcr_ is the cycle of PCR reaction, and *R*_designated_ is the ratio of designated reads (i.e., sequins reads) to all raw reads. The compositional data was normalized using total sum scaling (i.e., relative abundance). The abundance bias of each sequins component was calculated as the ratio between measured abundance and reference abundance, i.e., the theoretical value (for measuring the kit bias) or the mean value in non-WGA-based libraries (for measuring the WGA bias). Jensen-Shannon divergence was calculated using the philentropy R package v0.5.0 [32] and further square rooted to obtain Jensen-Shannon distance, which is a value between 0 and 1 with 0 denoting identical datasets.

Nanopore sequences were demultiplexed and trimmed for adaptors and barcodes using Porechop v0.2.4 (https://github.com/rrwick/Porechop). Kraken2 v2.1.2 was used for the taxonomic classification at the species level. Besides, the clean reads were mapped to the genome of *Homo sapiens* and 13 most abundant bacterial species that in total accounted for more than 90% of the microbial reads using Minimap2 v2.23 [33]. Reads having supplementary alignments (SAM flags 2048 and 2064) were filtered out, and the lengths of reads mapped to *A. baumannii* and *C. striatum* were summarized using SeqKit v0.10.2.

### Application of decontamination methods

Decontam v1.10.0 [17] was used based on the negative correlation between taxa abundance and DNA input amount (frequency mode), the prevalence (presence/absence across samples) of taxa in true positive samples versus negative controls (prevalence mode), or *either* mode. Forty thresholds increasing from 0.1 to 0.5 were tested (Additional file 2: Fig. S5a-e), and 0.5 was used for the frequency mode for all kits, whereas 0.5, 0.5, 0.5, 0.41, and 0.27 were used for the prevalence mode for Tn5_V, End_N, End_Q, Son_N, and Son_Q, respectively. SourceTracker v1.0.1 [18] was used by rarefying all libraries to 0.5 million reads and defining negative controls as the only known source of contaminants. For the 5/tenfold-NC method, the NC reference for each library construction method was calculated by averaging the relative abundances of each taxon in 2–3 NC samples. For the fivefold-NC method, a taxon that had an abundance greater than fivefold that of the NC reference was classified as a true component; otherwise, the taxon was classified as a contaminant, so was the tenfold-NC method. The performance of methods was evaluated on a per-read basis.

### Statistical analysis

Comparisons between the two groups were performed using the Wilcoxon tests with libraries from the same input amount as matched pairs. PERMANOVA was performed using the adonis2 function of vegan R package v2.5.7 [34]. Differential genera were identified using the DESeq2 R package v1.30.1 [35]. Multiple comparisons were corrected using the Benjamini–Hochberg false discovery rate algorithm [36] with a significance level of 0.05 (p.adj value).

## Supplementary Information

**Additional file 1:** **Table S1.** Composition and sequences of the synthetic DNA material sequins. **Table S2.** Characteristics of library construction kits. **Table S3.** 495 core microbial contaminations at the genus level.

**Additional file 2:** **Fig. S1.** Sequencing depth and rarefaction curves. **Fig. S2.** Performance of WGA in metagenomics using sub-nanogram DNA. **Fig. S3.** Comparison of non-WGA-based DNA library preparation methods. **Fig. S4.** Characteristics of contaminating DNA. **Fig. S5.** Thresholds and performance of *in silico* decontamination methods.

## Data Availability

All data generated or analyzed during this study are included in this published article, its supplementary information files, and publicly available repositories. The metagenomic data reported in this paper have been deposited in the Genome Sequence Archive in the National Genomics Data Center [37], Beijing Institute of Genomics (China National Center for Bioinformation), Chinese Academy of Sciences, under accession number CRA006267 [38].

## Abbreviations

WGA: Whole genome amplification
Son_N: Nugen Ovation Ultralow System V2 Kit
Son_Q: QIAGEN QIAseq Ultralow Input Library Kit
End_N: NEBNext Ultra II FS DNA Library Prep Kit
End_Q: QIAGEN QIAseq FX DNA Library Kit
Tn5_V: Vazyme TruePrep DNA Library Prep Kit
JSD: Jensen-Shannon distance
PCoA: Principal coordinates analysis
