# Supplementary material for: Toward efficient and high-fidelity metagenomic data from sub-nanogram DNA: evaluation of library preparation and decontamination methods
Source: BMC Biol. 2022 Oct 8;20:225. doi: 10.1186/s12915-022-01418-9 (PMC9548135; doi:10.1186/s12915-022-01418-9)
Supplement: Supplementary file 2 — Additional file 2: Fig. S1. Sequencing depth and rarefaction curves. Fig. S2. Performance of WGA in metagenomics using sub-nanogram DNA. Fig. S3. Comparison of non-WGA-based DNA library preparation methods. Fig. S4. Characteristics of contaminating DNA. Fig. S5. Thresholds and performance of in silico decontamination methods. [file 12915_2022_1418_MOESM2_ESM.docx]

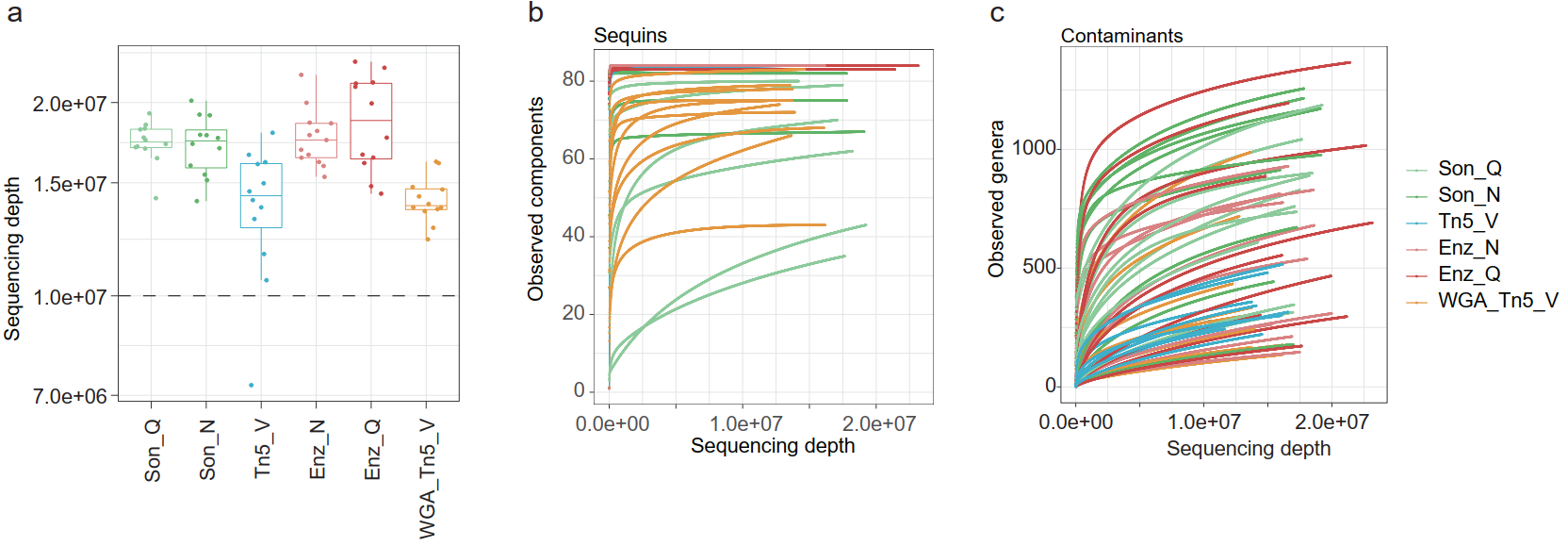


**Supplementary Figure 1.** **Sequencing depth and rarefaction curves.**

(a) Sequencing depth of each sample. (b-c) Rarefaction curves of observed sequins components (b) and observed contaminating genera (c).


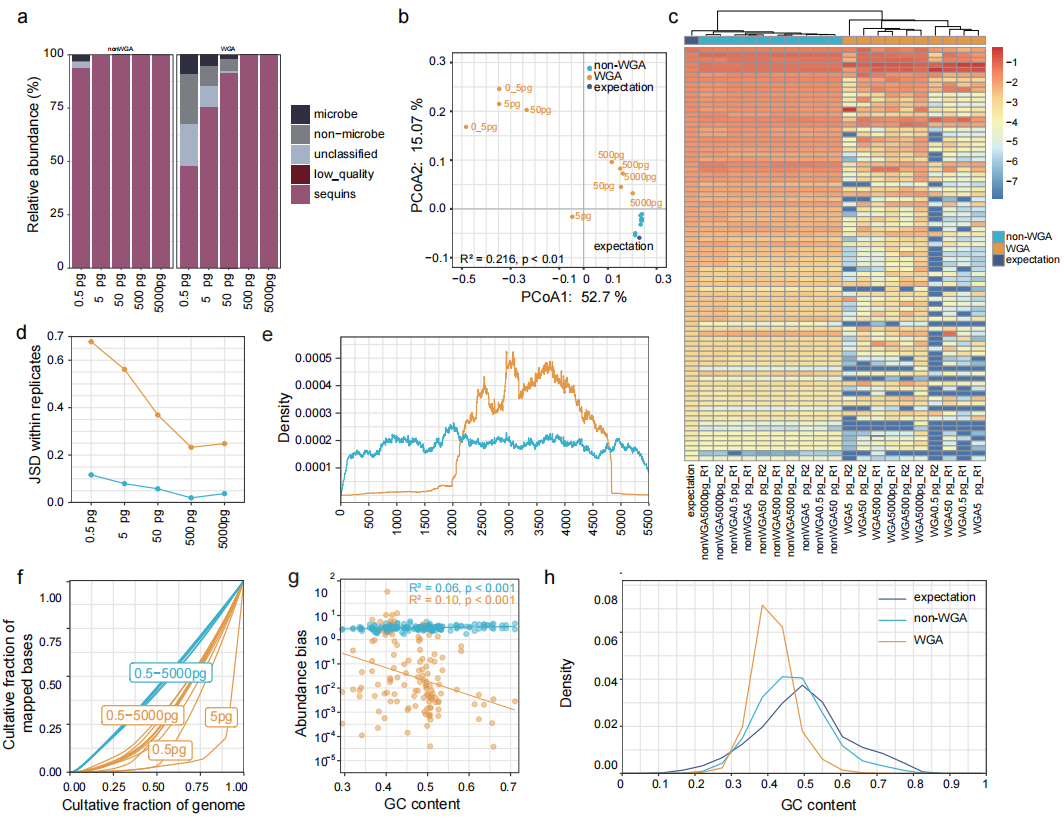


**Supplementary Figure 2. Performance of WGA in metagenomics using sub-nanogram DNA.**

(a) Reads composition of raw data. (b) PCoA plot based on JSD of sequins compositions. R^2^ and p values from PERMANOVA are shown. (c) Heatmap showing relative abundances of 83 sequins components. Ward.D2 clustering of samples is shown. (d) JSD between sequins compositions of experimental replicates. (e) Sequencing coverage of the most abundant component of sequins (S1081_ML_002_A) in libraries with 0.5 pg input.  (f) Lorenz curves of the most abundant component of sequins (S1081_ML_002_A). (g) Correlations between the abundance bias of sequins components and corresponding GC contents. R^2^ and p values from linear regression models are shown. (h) GC distribution of sequencing reads.


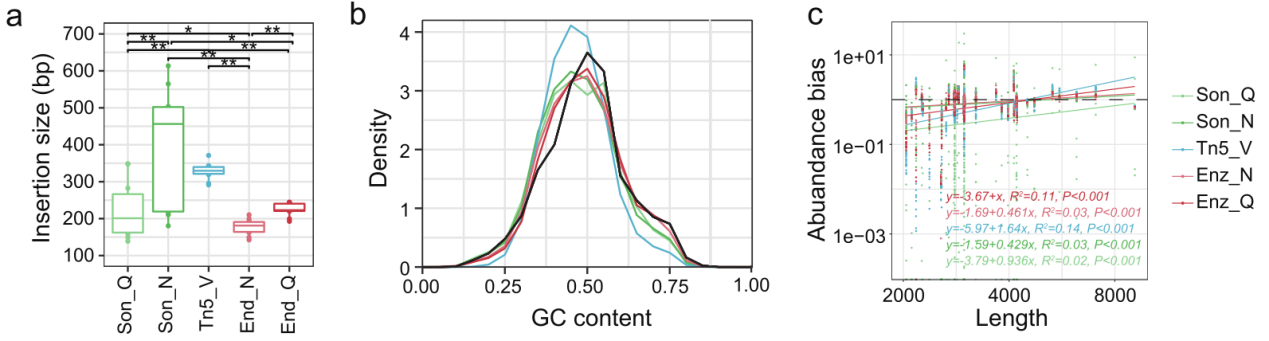


**Supplementary Figure 3. Comparison of non-WGA-based DNA library preparation methods.**

(a) Insertion size of libraries. *p < 0.05, **p < 0.01, Wilcoxon tests. (b) GC distribution of sequencing reads. The expected value is indicated by a black curve. (c) Correlations between the abundance bias of sequins components and corresponding fragment lengths. R^2^ and p values from linear regression models are shown.


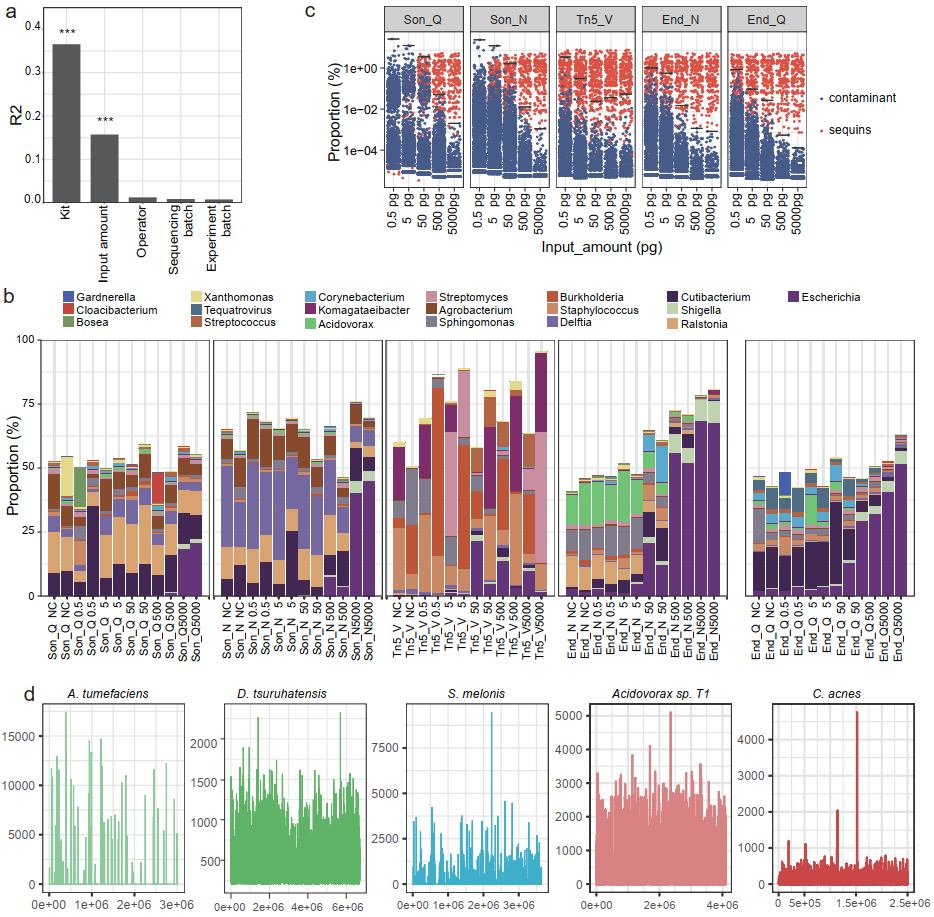


**Supplementary Figure 4. Characteristics of contaminating DNA.**

(a) PERMANOVA analysis of factors associated with the microbial contaminations. ***p < 0.001. (b) Relative abundances of top 19 contaminating genera. (c) Relative abundances of all sequins components (blue dots) and contaminating genera (red dots). Horizontal lines indicate the relative abundances of the most abundant contaminating genera in libraries with each input amount. (d) Genome coverage of *Agrobacterium tumefaciens* for Son_Q, *Delftia tsuruhatensis* for Son_N, *Sphingomonas melonis* for Tn5_V, *Acidovorax* sp. T1 for End_N, and *Cutibacterium acnes* for End_Q, which represented the most abundant contaminating taxa from each kit.


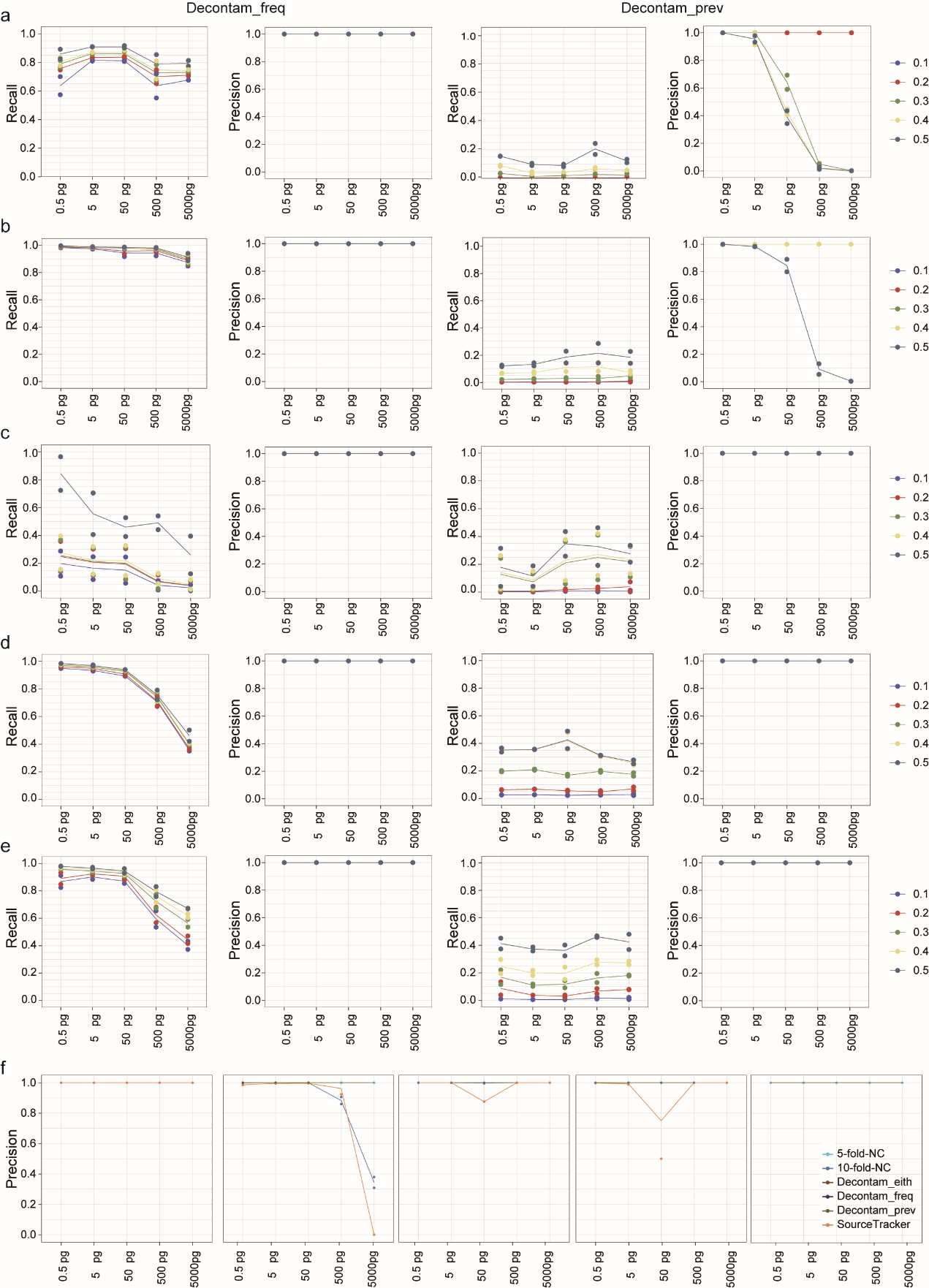


**Supplementary Figure 5. Thresholds and performance of *in silico* decontamination methods.**

(a-e) The recall and precision of Decontam-frequency with different thresholds, and the recall and precision of Decontam-prevalence with different thresholds shown from left to right for Son_Q (a), Son_N (b), Tn5_V (c), End_N (d), and End_Q (e). (f) The precision of different methods in identifying contaminations.
